# Oxidation of HMGB1 Causes Attenuation of Its Pro-Inflammatory Activity and Occurs during Liver Ischemia and Reperfusion

**DOI:** 10.1371/journal.pone.0035379

**Published:** 2012-04-13

**Authors:** Anding Liu, Haoshu Fang, Olaf Dirsch, Hao Jin, Uta Dahmen

**Affiliations:** 1 Experimental Transplantation Surgery, Department of General, Visceral and Vascular Surgery, Friedrich-Schiller-University Jena, Jena, Germany; 2 The Centre for Molecular Medicine, Shaoxing People's Hospital, the First Affiliated Hospital of Shaoxing University, Shaoxing, PR China; 3 Department of General, Visceral and Transplantation Surgery, University Hospital Essen, University of Duisburg and Essen, Essen, Germany; 4 Institute for Pathology, University Hospital of Jena, Jena, Germany; Universität Würzburg, Germany

## Abstract

High mobility group box 1 (HMGB1) is a nuclear transcription factor. Once HMGB1 is released by damaged cells or activated immune cells, it acts as danger molecule and triggers the inflammatory signaling cascade. Currently, evidence is accumulating that posttranslational modifications such as oxidation may modulate the pro-inflammatory potential of danger signals. We hypothesized that oxidation of HMGB1 may reduce its pro-inflammatory potential and could take place during prolonged ischemia and upon reperfusion.

Liver grafts were cold preserved for 24 h and flushed with saline in hourly intervals to collect the effluent. Liver grafts, cold-preserved for 6 h, were transplanted into syngeneic recipients to obtain serum and liver samples 24 h after initiation of reperfusion. Addition of the effluent to a macrophage culture induced the synthesis of tumor necrosis factor-alpha (TNF-α) and interleukin (IL)-6. The stimulatory activity of graft effluent was reduced after depletion of HMGB1 via immunoprecipitation. Oxidation of the effluent HMGB1 using H_2_O_2_ attenuated its stimulatory activity as well. Liver transplantation of cold preserved grafts caused HMGB1 translocation and release as determined by immunohistochemistry and ELISA-assay, respectively. Using Western blot with non-reducing conditions revealed the presence of oxidized HMGB1 in liver samples obtained after 12 h and in effluent samples after 16 h of cold preservation as well as in liver and serum samples obtained 24 h after reperfusion.

These observations confirm that post-translational oxidation of HMGB1 attenuates its pro-inflammatory activity. Oxidation of HMGB1 as induced during prolonged ischemia and by reoxygenation during reperfusion in vivo might also attenuate its pro-inflammatory activity. Our findings also call for future studies to investigate the mechanism of the inhibitory effect of oxidized HMGB1 on the pro-inflammatory potential.

## Introduction

Liver transplantation is currently the only efficient treatment for patients suffering from both acute and chronic liver disease. Although significant progress in liver transplantation has been made, primary graft dysfunction remains a problem. Injury inflicted on the graft may result from harvesting, ischemia, preservation and reperfusion.

Cold ischemia is an independent risk factor for primary dysfunction and delayed graft function [1]. Cold ischemia activates hepatic sinusoidal epithelial cells and Kupffer cells to produce inflammatory cytokines and to express adhesion molecules [2], [3]. Activation of the inflammatory response results in hepatic damage upon liver transplantation. Activation of the inflammatory cascades involves the release of danger signals from injured cells and activated immune cells [4]. Up to now, an array of danger signals has been identified with high mobility group box 1 (HMGB1) being the most widely explored [5]–[7].

HMGB1 was initially defined as a nuclear protein which loosely binds to chromatin, and plays a pivotal role to bending DNA and regulating transcription [8]–[10]. Recent studies have demonstrated that HMGB1 also acts as a mediator of inflammation when passively released by necrotic cells and actively secreted by immune cells [8], [11]–[14]. Immune cells, such as monocytes and macrophages stimulated by lipopolysaccharide (LPS), tumor necrosis factor-alpha (TNF-α) or interleukin (IL)-1 secrete HMGB1 [11]. Serum HMGB1 levels are increased after endotoxin exposure, and administration of anti-HMGB1 antibody protects mice from LPS-induced death [11]. Serum levels of HMGB1 are also elevated in patients with inflammatory diseases, such as pancreatitis, arthritis, and hemorrhagic shock [15]. Recent reports demonstrated that HMGB1 acts as an alarmin initiating the inflammatory response resulting from ischemia/reperfusion (I/R) injury in several organs, including liver, kidney, heart and brain [16]–[21].

HMGB1 can be subject to different types of post-translational modification, including acetylation [22], phosphorylation [23], methylation [24] and oxidation [25]. Recently, it is reported that the immune response mediated by HMGB1, lies not simply in the release of HMGB1, but rather in the oxidative state of the released HMGB1 [26], [27]. We demonstrated previously that HMGB1 was released into the effluent during prolonged cold saline preservation of liver grafts [28]. We also demonstrated that transplantation of a cold-preserved liver graft did not interfere with spontaneous graft acceptance [29]. These data indicate that HMGB1-release did not result in continuing inflammatory injury in this setting.

In the present study, we aimed to test these hypotheses that oxidation of HMGB1 may attenuate its inflammatory potential and could take place during prolonged ischemia and liver transplantation.

## Results

### Graft effluent induces pro-inflammatory cytokine synthesis by cultured rat peritoneal macrophages

**Figure 1:**
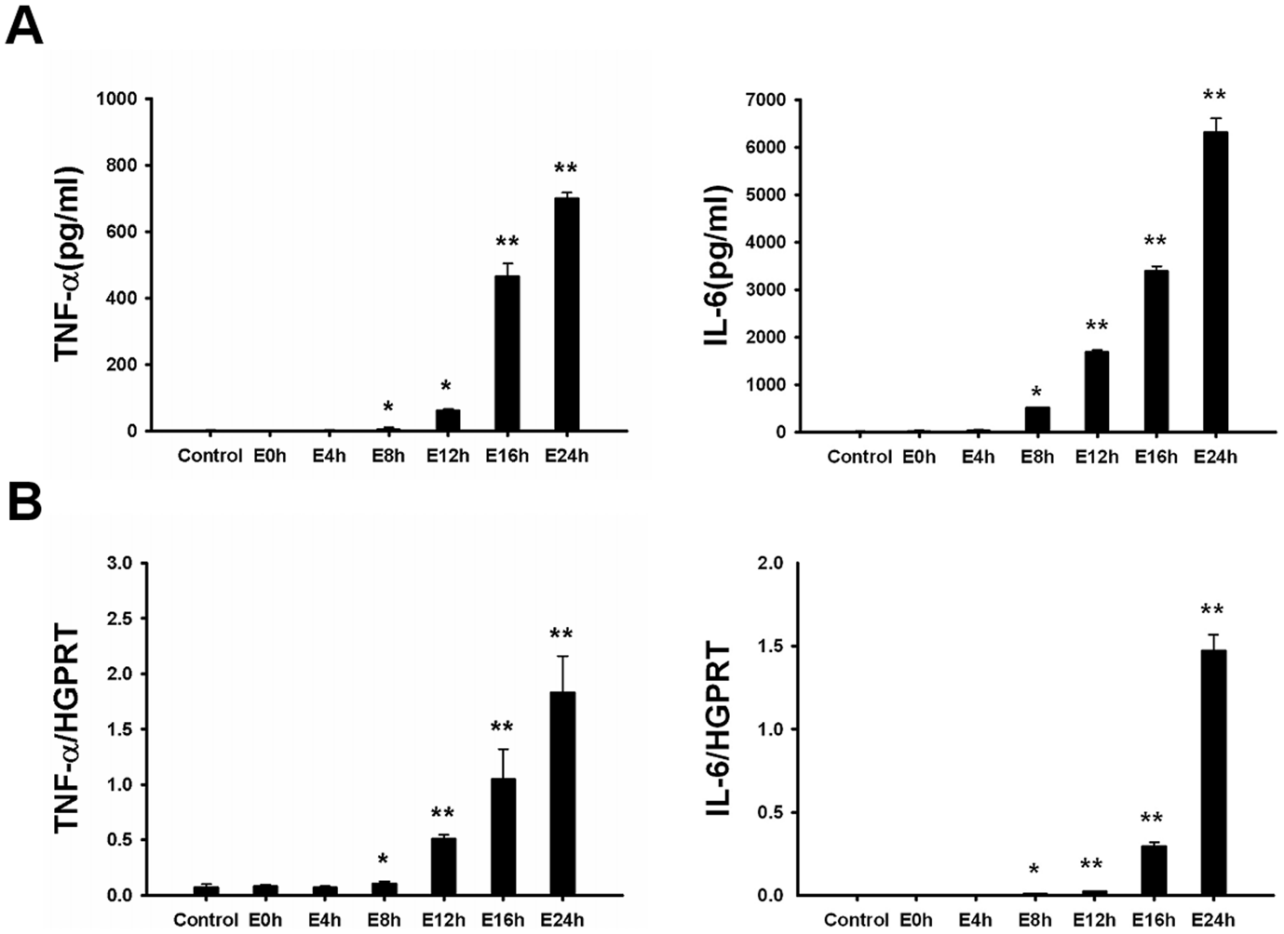
Graft effluent stimulates the synthesis of inflammatory cytokines in peritoneal macrophages. (A) Macrophages were stimulated with effluent (100 µl) and the supernatants were examined for levels of inflammatory cytokines (TNF-α and IL-6) by ELISA 6 h after stimulation. (B) Quantitative PCR was performed to determine the mRNA expression levels of TNF-α and IL-6. TNF-α and IL-6 concentration in the supernatants, as well as intracellular TNF-α and IL-6 mRNA levels were significantly increased after stimulation with effluent obtained after 8 h of cold storage. Data are shown as mean ± SD. *p<0.05, **p<0.001. E, effluent; TNF-α, tumor necrosis factor-alpha; IL, interleukin; HGPRT, hypoxanthine-guanine phosphoribosyltransferase; ELISA, enzyme-linked immunosorbent assay; PCR, polymerase chain reaction.

TNF-α and IL-6 are known to increase in serum and liver tissue upon reperfusion and play pivotal role in the pathophysiology of hepatic I/R injury [30], [31]. To observe whether graft effluent may induce TNF-α and IL-6 synthesis by macrophages, effluent (100 µl) was added to cultured peritoneal macrophages for 6 h. Subsequently, the levels of both inflammatory cytokines, TNF-α and IL-6, in the supernatants and their mRNA expression in macrophages were measured. There was no significant increase in mRNA levels or secreted protein levels for TNF-α or IL-6 by macrophages cultured with effluent collected at 0 h or 4 h after cold preservation of livers. In contrast, effluent collected at 8 h, 12 h, 16 h or 24 h after cold preservation induced significant and rising increases of both protein and mRNA levels for TNF-α and IL-6 (p<0.05) (Fig. 1).

### The stimulatory activity of graft effluent is attenuated after the depletion of HMGB1

**Figure 2:**
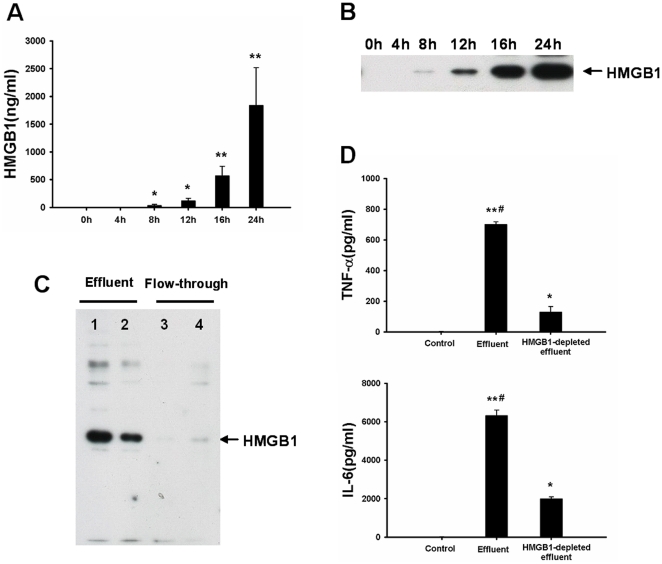
Pro-inflammatory activity of graft effluent is decreased after HMGB1 depletion. HMGB1 concentration in effluent was detected by ELISA-assay (A) or western blot (B), respectively. Effluent HMGB1 was significantly increased as early as 8 h and then upregulated in a time-dependent manner up to 24 h (*p<0.001, **p<0.0001 vs 0 h). (C) The concentration of HMGB1 in effluent obtained after 24 h of cold storage was drastically reduced after HMGB1 depletion using immunoprecipitation. Lane 1–2 showed the HMGB1 in effluent. Lane 3–4 displayed the HMGB1 in flow-through after HMGB1 depletion. (D) Rat peritoneal macrophages were cultured in the presence of effluent (100 µl), or HMGB1-depleted effluent (flow-through; 100 µl) for 6 h. The inflammatory activity of effluent was markedly decreased after HMGB1 depletion when compared with complete effluent. The experiment was performed in triplicates with similar results. Data are shown as mean ± SD. *p<0.001, **p<0.0001 vs blank control; # p<0.001 vs HMGB1-depleted effluent. HMGB1, high mobility group box 1; TNF-α, tumor necrosis factor-alpha; IL, interleukin.

We previously reported that HMGB1 was released into effluent during cold ischemia [28]. In the present study, HMGB1 levels in the effluent were negligible at 0 h (1.5±0.4 ng/ml) and became detectable as early as 8 h (36±19 ng/ml), and then increased in a time-dependent manner up to 24 h (1824±680 ng/ml) (Fig. 2A). These results were confirmed by western blot analysis showing that the amount of HMGB1 present in effluent was elevated after 8 h cold ischemia (Fig. 2B). Effluent with the highest concentration of HMGB1, obtained after 24 h of cold storage, was selected for the HMGB1 pro-inflammatory activity assay.

To determine whether effluent HMGB1 was biologically active as a pro-inflammatory mediator, HMGB1 was depleted from effluent using immunoprecipitation prior to stimulation, as shown in Fig. 2C. The synthesis of TNF-α and IL-6 was reduced by about 60% when using effluent depleted of HMGB1 (Fig. 2D) in comparison to complete effluent. This finding indicated that this response was mainly mediated by effluent HMGB1.

### Oxidation of HMGB1 attenuates its stimulatory activity in cultured macrophages

**Figure 3:**
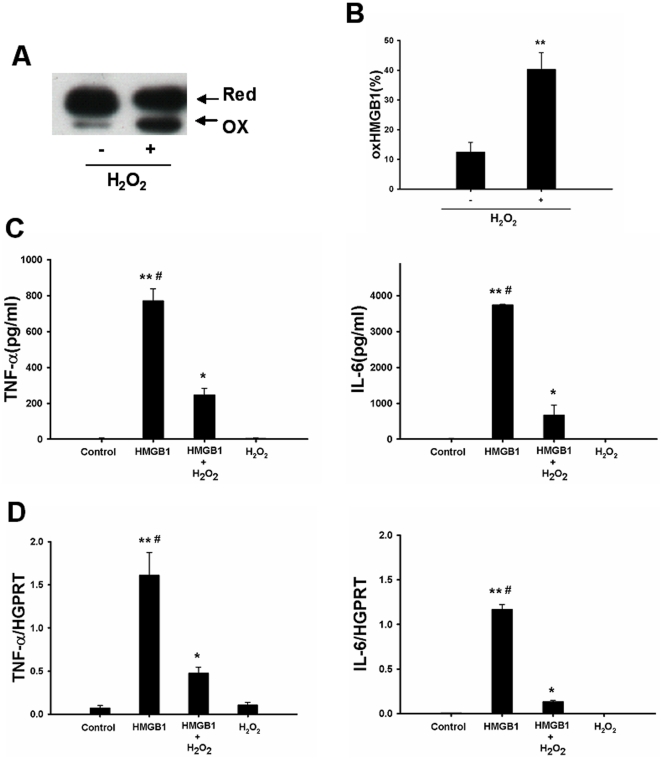
HMGB1 inflammatory activity is attenuated after oxidation in vitro. (A) HMGB1 was firstly extracted from effluent obtained after 24 h of cold storage and then treated with H_2_O_2_ (50 µM) for 1 h on ice. Oxidized HMGB1 was separated on a non-reducing SDS-PAGE gel and detected by western blot with a polyclonal anti-HMGB1 antibody. (B) The gray value of bands was calculated by ImageJ. The relative amount of oxidized HMGB1 was expressed by oxidized HMGB1/total HMGB1. Macrophages were stimulated with effluent HMGB1 (0.5 µg/ml) or oxidized HMGB1 (effluent HMGB1 pretreated with H_2_O_2_; 0.5 µg/ml) for 6 h. TNF-α and IL-6 concentration in the supernatants (C), as well as intracellular TNF-α and IL-6 mRNA levels (D) were then determined. Effluent HMGB1 pretreated with H_2_O_2_ significantly attenuated its inflammatory activity. The experiment was performed in triplicates with similar results. Data are shown as mean ± SD. *p<0.001, **p<0.0001 vs blank control; # p<0.001 vs HMGB1+H_2_O_2_ group. HMGB1, high mobility group box 1; TNF-α, tumor necrosis factor-alpha; IL, interleukin; HGPRT, hypoxanthine-guanine phosphoribosyltransferase; Ox, oxidized; Red, reduced.

To observe whether oxidized HMGB1 could neutralize its stimulatory activity, HMGB1 was firstly extracted from effluent obtained after 24 h of cold storage and oxidation of HMGB1 was induced by pretreatment of the effluent HMGB1 with H_2_O_2_ prior to stimulation. Oxidation of HMGB1 can induce a mobility shift in non-reducing SDS-PAGE [25]. Immunoblotting showed that the amount of oxidized HMGB1 was dramatically increased after treatment with H_2_O_2_ (Fig. 3A–B). As shown in Fig. 3C–D, The synthesis of TNF-α and IL-6 was reduced by approximately 80% when using effluent HMGB1 pretreated with H_2_O_2_. This finding indicated that inhibition of the pro-inflammatory activity was mainly mediated by oxidation of HMGB1.

### Oxidation of HMGB1 attenuates its stimulatory activity in vivo

**Figure 4:**
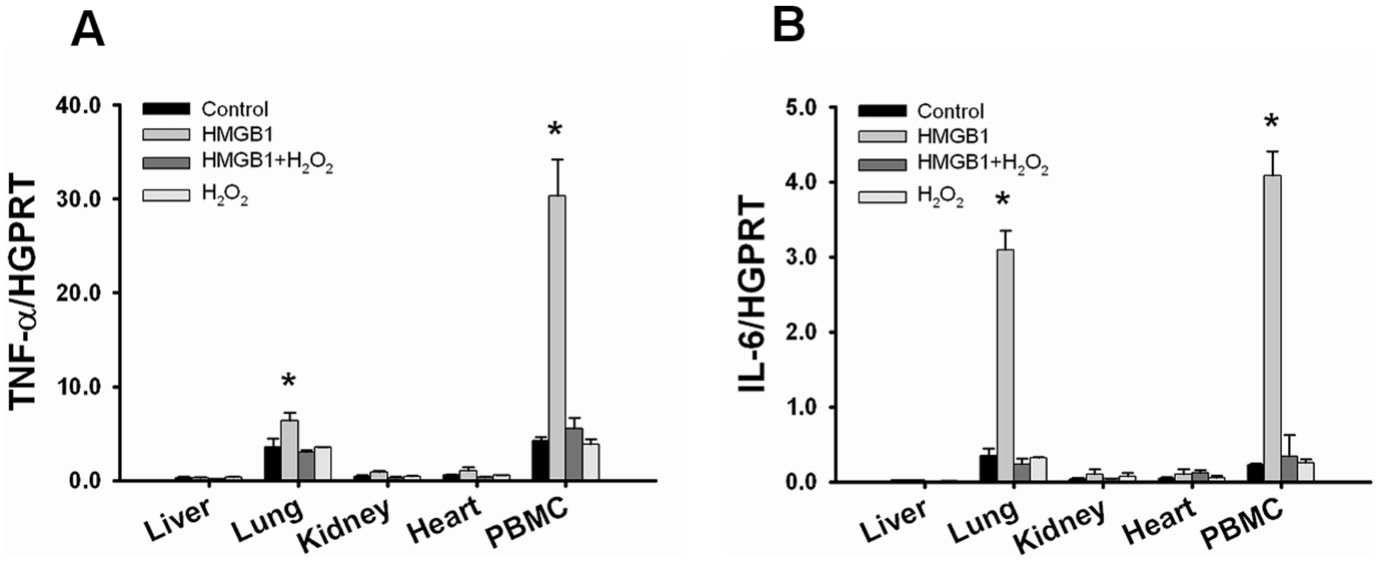
HMGB1 inflammatory activity is attenuated after oxidation in vivo. Recombinant HMGB1 (rHMGB1) was pretreated with 50 µM H_2_O_2_ for 1 h on ice. TNF-α mRNA and IL-6 mRNA expression levels were measured by quantitative PCR in liver, lung, heart, kidney and PBMC 6 h after injecting either unmodified (rHMGB1; 350 µg/rat) or oxidized HMGB1 (rHMGB1 pretreated with H_2_O_2_; 350 µg/rat) to naive rats. mRNA levels for TNF-α (A) and IL-6 (B) were significantly elevated in lung and PBMC after administration of rHMGB1. In contrast, rHMGB1 pretreated with H_2_O_2_ did not upregulate the expression of TNF-α and IL-6. Data are shown as mean ± SD. *p<0.001 vs HMGB1+H_2_O_2_ group. HMGB1, high mobility group box 1; TNF-α, tumor necrosis factor-alpha; IL, interleukin; HGPRT, hypoxanthine-guanine phosphoribosyltransferase; PBMC, peripheral blood mononuclear cells; PCR, polymerase chain reaction.

It was reported that administration of recombinant HMGB1 (rHMGB1) to mice induced sepsis and acute lung inflammation [11], [32]. Interestingly, administration of rHMGB1 (1.2 mg/kg) to naive rats induced mRNA expression of TNF-α and IL-6 in lung and PBMC, but not in liver, kidney and heart. We studied whether administration of H_2_O_2_-oxidized HMGB1 could attenuate its inflammatory activity in vivo. Indeed, the HMGB1-mediated effect on the expression of inflammatory cytokines in lung and PBMC was reduced when animals were treated with rHMGB1 subjected to H_2_O_2_ (Fig. 4).

### HMGB1 expression is increased after liver transplantation

**Figure 5:**
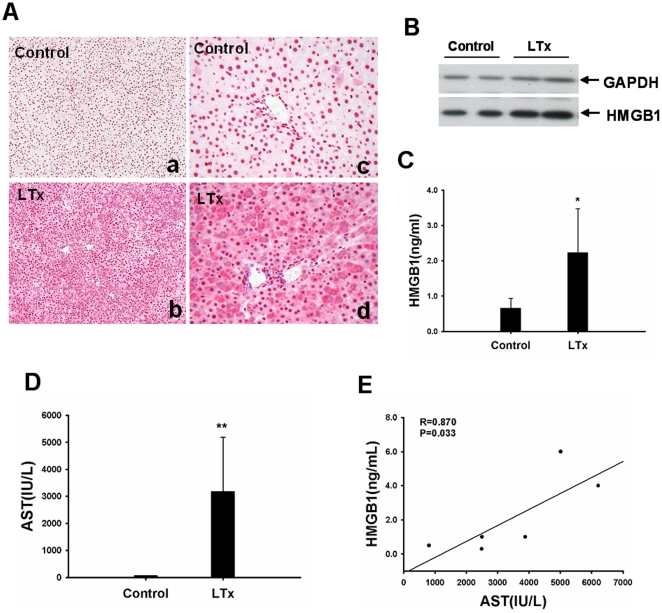
Liver transplantation causes HMGB1 translocation and release. (A) Immunohistochemical staining of HMGB1 demonstrated that HMGB1 was translocated from nucleus to cytoplasm in hepatocytes after liver transplantation (a–b, original magnification ×100; c–d, original magnification ×200). Representative images from six rats/group were selected. (B) Expression levels of HMGB1 protein in grafts were significantly increased after liver transplantation. The results were obtained using liver homogenates from six individual animals. (C) Serum concentrations of HMGB1, as measured by ELISA were significantly increased 24 h after liver transplantation. (D) Liver transplantation caused a severe hepatocellular injury as indicated by higher release of AST. (E) Serum HMGB1 levels positively correlated with AST levels in the serum. Data are shown as mean ± SD. *p<0.05, **p<0.001 vs normal control rats. HMGB1, high mobility group box 1; AST, aspartate aminotransferase; GAPDH, glyceraldehyde-3-phosphate dehydrogenase; LTx, liver transplantation; ELISA, enzyme-linked immunosorbent assay.

As shown in Fig. 5A, liver transplantation resulted in HMGB1 translocation from the nucleus to the cytoplasm in hepatocytes. More than 50% of hepatocytes with cytoplasmic HMGB1 staining were recorded after 24 h reperfusion. HMGB1 protein was upregulated by liver cells, accounting for an approximately twofold increase at 24 h after liver transplantation (Fig. 5B). Liver transplantation also caused HMGB1 release. Serum HMGB1 levels were elevated by approximately fourfold at 24 h as quantified using an ELISA-assay (Fig. 5C). Of note, serum HMGB1 levels correlated with AST levels at 24 h after liver transplantation (r = 0.870, p = 0.033; Fig. 5E). This finding suggests that liver transplantation upregulated HMGB1 expression and serum HMGB1 was mainly released by damaged hepatocytes.

### Oxidation of HMGB1 occurs after prolonged cold storage of the liver and after liver transplantation

**Figure 6:**
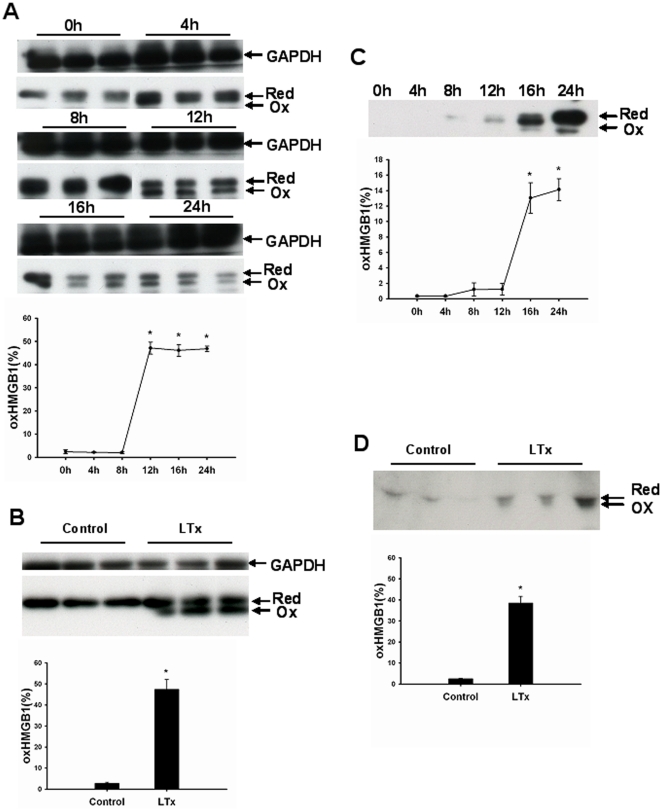
Oxidized HMGB1 is produced after prolonged cold ischemia and reperfusion in liver transplantation. (A–B) Total hepatic proteins (10 µg) were separated on a non-reducing SDS-PAGE gel and HMGB1 protein was detected by western blot. The gray value of bands was calculated by ImageJ. The relative amount of oxidized HMGB1 was expressed by oxidized HMGB1/total HMGB1. Oxidized HMGB1 was produced in liver graft during cold storage (A) and 24 h after transplantation (B) Each lane represented a separate animal. The results were obtained using liver homogenates from six individual animals per each observation time point. Data are shown as mean ± SD. *p<0.001 vs 0 h. Effluent (C) or serum proteins (D) (10 µl) were separated on a non-reducing SDS-PAGE gel. The gray value of bands was calculated by ImageJ. The relative amount of oxidized HMGB1 was expressed by oxidized HMGB1/total HMGB1. Oxidized HMGB1 was found in effluent after 16 h cold ischemic storage of liver and in serum obtained 24 h after transplantation. The results shown are representative of six animals/group. Data are shown as mean ± SD. *p<0.001 vs normal control rats. HMGB1, high mobility group box 1; GAPDH, glyceraldehyde-3-phosphate dehydrogenase; Ox, oxidized; Red, reduced; Con, normal control; LTx, liver transplantation.

To determine whether HMGB1 was oxidized after prolonged cold ischemia and liver transplantation, effluent proteins, hepatic proteins or serum proteins were firstly separated on non-reducing SDS-PAGE and HMGB1 protein was detected by immunoblotting. As shown in Fig. 6, oxidized HMGB1 was detected in cold preserved ischemic liver grafts (Fig. 6A) and in the effluent obtained from those livers (Fig. 6C). Total HMGB1 was slightly increased 4 h after explantation, reached a maximum between 8 h and 12 h of cold storage and decreased thereafter, possibly related to the loss of HMGB1 into the effluent. Oxidized HMGB1 was significantly increased in liver samples obtained after 12 h and in effluent samples after 16 h of cold preservation. Band sizes for oxidized and reduced fraction of HMGB1 in liver tissue were of similar thickness, whereas the fraction of oxidized HMGB1 in the effluent was much smaller. We also detected oxidation of HMGB1 in the liver graft tissue (Fig. 6B) and serum (Fig. 6D) of rats 24 h after liver transplantation, suggesting a reduction of its inflammatory potential after ischemia and reperfusion.

## Discussion

HMGB1 has been proposed to induce the inflammatory response in hepatic I/R injury but its role in this process is not fully understood. Furthermore, HMGB1 is often post-translationally modified [7], which might affect its function. In this study, we demonstrated that: (1) HMGB1, released during cold ischemia, induced the expression of inflammatory mediators by cultured macrophages; (2) oxidation of HMGB1 using H_2_O_2_ reduced its pro-inflammatory potential both in vitro and in vivo; and (3) oxidation of HMGB1 took place during prolonged cold ischemia and reperfusion in liver transplantation.

HMGB1 is released from ischemic liver cells and has been shown to mediate the inflammation and injury in liver I/R injury [16], [20]. In the present study, we demonstrated that cold storage of the liver graft and liver transplantation could induce HMGB1 translocation and release. This finding is consistent with that of Ilmakunnas et al. They reported that HMGB1 levels peaked 10 min after reperfusion and began to decrease progressively within 1 to 2 hours after reperfusion [33]. Effluent HMGB1 obtained from liver grafts during cold ischemia significantly upregulated the expression of inflammatory mediators by cultured macrophages. This finding provides direct evidence that HMGB1 can participate in the meditation of inflammation in hepatic I/R injury. HMGB1 released from damaged liver cells, may exert the same effect and cause the synthesis of pro-inflammatory cytokine in livers subjected to I/R injury in vivo. This hypothesis is supported by a previous observation from Tsung et al. [16]. In their model, HMGB1 levels were increased during liver I/R as early as 1 h after reperfusion, and neutralizing antibody to HMGB1 decreased production of TNF-a, IL-6 and inducible NO synthase (iNOS) in hepatic warm I/R.

HMGB1 contains three cysteines, Cys23, 45, and 106. Cys23 and 45 induce conformational changes in response to oxidative stress, whereas Cys106 is critical for HMGB1 translocation from nucleus to cytoplasm [25]. We previously demonstrated that HMGB1 was translocated from the nucleus to the cytoplasm in hepatocytes as early as 4 h after cold ischemia [28]. In the present study, we observed that oxidation of HMGB1 occurred upon prolonged cold ischemia in parallel with its translocation into cytoplasm and the release into the effluent. Oxidation of HMGB1 was also present in liver tissue and serum obtained 24 h after reoxygenation. In contrast, oxidized HMGB1 was undetectable in normal liver tissue. Our observations support the findings of Ilmakunnas and Sahu [33], [34], demonstrating that HMGB1 is present partially in an oxidized form during liver I/R.

We were interested to elucidate whether oxidation of HMGB1 could attenuate its inflammatory potential. For oxidation or inhibition purposes, HMGB1 was incubated for 1 h on ice with H_2_O_2_ at a concentration of 50 µM, which is normally found within ischemic/reperfused liver tissue [35]. One limitation to this experiment is that treatment with H_2_O_2_ may oxidize HMGB1 not only Cys residues but also at other regions which needed for its stimulatory function. First we demonstrated that oxidation of effluent HMGB1 with H_2_O_2_ reduced the inflammatory response in the macrophage bioassay in vitro. This observation is supported by the results from other groups [26], [36]. The reduced inflammatory activity may be caused by the oxidation of Cys106 within the HMGB1 molecule. Kazama et al. reported that oxidation of Cys106 is necessary to block the stimulatory activity of HMGB1 [26]. Cys106 has been recently implicated in the binding of HMGB1 to macrophages toll-like receptor 4 [36]. Yang and colleagues observed that other alterations such as the mutation of Cys106 also prevented the HMGB1-induced activation of and cytokine release by cultured macrophages [36].

To determine whether oxidation of HMGB1 could attenuate the inflammatory response also in vivo, we injected rHMGB1 as well as oxidized rHMGB1 and observed the mRNA expression of pro-inflammatory cytokines in different organs. Abraham et al. reported that administration of rHMGB1 to mice led to an acute inflammatory injury of the lungs with neutrophil accumulation, the development of lung edema and increased pulmonary production of inflammatory cytokines [32]. Application of lower doses HMGB1 in vivo (10 to 50 µg/mouse) caused similar symptoms as observed in animals with systemic inflammatory syndrome e.g. after endotoxemia. High doses of HMGB1 (i.e., 500 µg/mouse) were lethal for mice [11]. We used a dose of 350 µg/rat (1.2 mg/kg), which is in a similar range as used by others in mice. The inflammatory response, as measured by TNF-α and IL-6 mRNA expression, was significantly increased in lung and PBMC, but not in other organs 6 h after HMGB1 administration. In contrast, injection of oxidized HMGB1 attenuated the expression of both cytokines in both lung and PBMC. Taken together, oxidation of HMGB1 reduced its inflammatory activity both in vitro and in vivo.

However, liver I/R injury may also induce other posttranslational modifications of HMGB1. Unmodified HMGB1, or other forms of HMGB1, such as acetylated, phosphorylated and methylated HMGB1 may also exist in extracellular space. These forms of HMGB1 have stimulatory activity [8], [12], [14] and may trigger the inflammation in liver I/R injury [16]. Thus, although oxidized HMGB1 is produced upon reperfusion and attenuates its pro-inflammatory activity, other forms of HMGB1 that are present in such settings are likely to override the inhibitory effects of oxidized HMGB1 during liver I/R.

In summary, HMGB1 released into the effluent during prolonged cold saline preservation, contributed to the pro-inflammatory activity in a macrophage assay. Oxidation of HMGB1 as induced by H_2_O_2_-pretreatment attenuated its inflammatory activity both in vitro and in vivo. Oxidation of HMGB1 as induced during prolonged ischemia and by reoxygenation during reperfusion in vivo might also attenuate its pro-inflammatory activity.

These observations confirm that HMGB1 post-translational modifications are decisive for the pro-inflammatory activity. Our findings also call for future studies to investigate the mechanism of the inhibitory effect of oxidized HMGB1 on the pro-inflammatory potential in more depth.

## Materials and Methods

### Ethics statement

All animal procedures were carried out according to the German Animal Welfare Legislation. Animal experiments were approved by the Bezirksregierung Düsseldorf (Approval number: G893/06).

### Experimental design

The experiments were designed to investigate whether effluent HMGB1 may act as inflammatory cytokine and posttranslational oxidation of HMGB1-protein may attenuate its inflammatory potential. We also examined whether hepatic HMGB1 was oxidized during cold ischemia and reperfusion after liver transplantation.

To determine whether effluent HMGB1 could trigger the synthesis of pro-inflammatory cytokines by cultured macrophage, effluent was collected after 24 h of cold storage of liver grafts and effluent HMGB1 was depleted by immunoprecipitation. Peritoneal macrophages were cultured for 6 h alone or in the presence of effluent or effluent after depletion of HMGB1. TNF-α and IL-6 concentration in culture supernatants and their mRNA expression in macrophages were assayed by ELISA or quantitative PCR, respectively.

To determine whether oxidized HMGB1 could attenuate the synthesis of pro-inflammatory cytokines by cultured macrophages, HMGB1 was firstly extracted from effluent obtained after 24 h of cold storage by immunoprecipitation and then oxidized via pretreatment with H_2_O_2_. Peritoneal macrophages were cultured alone or in the presence of effluent HMGB1 or oxidized HMGB1 (effluent HMGB1 pretreated with H_2_O_2_). The synthesis of TNF-α and IL-6 was measured as described above.

To determine whether oxidized HMGB1 could attenuate its stimulatory activity in vivo, recombinant HMGB1 was oxidized via pretreatment with H_2_O_2_. Oxidized HMGB1 protein or HMGB1 protein was administered in a single dose by intraperitoneally (i.p.). Determination of TNF-α and IL-6 expression was used as indicator of the inflammatory response.

To determine whether oxidized HMGB1 was generated during cold ischemia and reperfusion in liver transplantation, oxidation of HMGB1 in liver tissue and effluent samples obtained during cold preserved liver grafts as well as in liver tissue and serum samples obtained 24 h after transplantation were examined.

### Animals

Male inbred Lewis rats, purchased from Central Animal Facility of the University Hospital Essen, weighing between 250∼300 g, were used for this study. All animals were housed under standard animal care conditions and had free access to water and rat chow ad libitum.

### Isolation and perfusion of rat liver

Livers were isolated and perfused by the method outlined by us in details earlier [28]. At specific time intervals, the livers were flushed with saline (4°C) at a constant pressure of 10 cm H_2_O through the portal vein and 1.5 ml effluent was collected from the infrahepatic vena cava. Samples were centrifuged thereafter at 4°C to remove erythrocytes.

### Preparation and treatment of effluent HMGB1

Effluent was collected at defined time-points and 100 µl effluent was used for treatment of macrophages. In defined experiments, HMGB1 was depleted from effluent obtained after 24 h of cold storage by immunoprecipitation analysis. Depletion of HMGB1 was verified by immunoblotting. Effluent HMGB1 was treated with 50 µM H_2_O_2_ for 1 h on ice. Oxidized HMGB1 was separated by non-reducing SDS-PAGE gel and detected by immunoblotting.

### Immunoprecipitation analysis

Crosslink immunoprecipitation kit was bought from Pierce (Thermo Pierce, Rockford, USA). Immunoprecipitation experiments were performed according to the instructions of the manufacturer. Briefly, HMGB1 antibody (10 µg; Abcam, Cambridge, UK) was firstly incubated with protein A/G plus agarose resin (20 µl), and then was covalently cross-linked onto protein A/G resin by 450 µM DSS at room temperature for 1 h. Normal rabbit IgG (Sigma-Aldrich, St.Louis, USA) was used as a negative control. Effluent obtained after 24 h of cold storage was firstly pre-cleared using the control agarose resin to reduce nonspecific protein binding. Precleared effluent was incubated with protein A/G beads coupling with anti-HMGB1 at 4°C overnight. The flow-through was collected by centrifugation for HMGB1 depletion experiments. Effluent HMGB1 was eluted from beads by using antigen elution buffer for pro-inflammatory activity analysis.

### Administration of HMGB1

Recombinant HMGB1 (rHMGB1) (Sigma-Aldrich, St.Louis, USA) was pretreated with 50 µM H_2_O_2_ for 1 h on ice. Oxidized HMGB1 protein or HMGB1 protein was administered in a single dose by intraperitoneally (i.p.) injection at a concentration of 350 µg per rat (1.2 mg/kg) in 1 ml of PBS, respectively. Control rats were given 50 µM H_2_O_2_ in 1 ml of PBS. Rats were scarified and livers, kidneys, lungs, hearts, blood sampling were harvested 6 h after the i.p. injection. Peripheral blood mononuclear cells (PBMC) were separated by Ficoll density gradient centrifugation (Biocoll; Biochrom AG, Berlin, Germany).

### Isolation and culture of peritoneal macrophages

Isolation and culture of peritoneal macrophages were performed as described previously [37]. Peritoneal macrophages were cultured alone or in the presence of effluent (100 µl), effluent depleted of HMGB1 (100 µl), effluent HMGB1 (0.5 µg/ml), or oxidized HMGB1 (effluent HMGB1 pretreated with H_2_O_2_; 0.5 µg/ml).

### Liver transplantation

Liver grafts were subjected to 6 h cold ischemia prior to the transplantation procedure based on the method described by Kamada [38]. Rats were sacrificed 24 h after transplantation. To assess hepatocellular injury following liver transplatation, aspartate aminotransferase (AST) was measured in serum using an Automated Chemical Analyzer (Bayer; Leverkusen, Germany).

### Gel electrophoresis and western blot

Ten µg total proteins or 10 µl effluent proteins were loaded per well and separated on reducing or non-reducing 12% gel by sodium dodecyl sulfate-polyacrylamide gel electrophoresis (SDS-PAGE) followed by western blot and staining with HMGB1 antibody (1∶1000; Abcam, Cambridge, UK) or GAPDH antibody (1∶20000; Sigma-Aldrich, St.Louis, USA). Signals were detected with Lumi-Light Western Blot Substrate (Roche, Mannheim, Germany) and exposed to high sensitivity films (GE Healthcare, Buckinghamshire, UK) for autoradiography. The gray value of bands was calculated by ImageJ 1.43 G (NIH, Bethesda, USA).

### HMGB1 immunohistochemistry

HMGB1 immunohistochemical analysis was performed as described previously [28]. After de-paraffinization and rehydration, antigen retrieval was performed using citrate-EDTA buffer (10 mM citric acid, 2 mM EDTA, 0.05% Tween 20, pH 6.2) for 20 mins at 100°C. Then sections were incubated with HMGB1 antibody (1∶500; Abcam, Cambridge, UK) for 1 h at room temperature, followed by detection using the PowerVision System (ImmunoLogic, Duiven, Netherlands). The percentage of hepatocytes with only cytoplasmic HMGB1 staining out of the total number of hepatocytes was calculated.

### Enzyme-linked immunosorbent assay (ELISA)

Effluent HMGB1 or serum HMGB1 was measured with a commercial ELISA-assay (Shino-Test, Kanagawa, Japan). IL-6 and TNF-α concentration in culture supernatants were measured using commercially available ELISA kits (R&D Systems Europe, Abingdon, UK). All procedures were performed according to the instructions of the manufacturers.

### Quantitative polymerase chain reaction (PCR)

Total RNA was extracted from liver tissue or cultured cells using the RNeasy kit (Qiagen, Hilden, Germany) according to the manufacturer's instruction. cDNA synthesis was performed using the First-Strand cDNA synthesis kit (Invitrogen, Carlsbad, USA). The sense, antisense primers and universal probe library (Roche Diagnostics GmbH, Mannheim, Germany) used for the analysis of the expression of TNF-α, IL-6 and hypoxanthine-guanine phosphoribosyltransferase (HGPRT) were as follows: TNF-α: 5′-TGAACTTCGGGGTGATCG-3′, 5′-GGGCTTGTCACTCGAGTTTT-3′ and probe #63; IL-6: 5′-CCTGGAGTTTGTGAAGAACAACT-3′, 5′-GGAAGTTGGGGTAGGAAGGA-3′ and probe #106; HGPRT: 5′-GACCGGTTCTGTCATGTCG-3′, 5′-ACCTGGTTCATCATCACTAATCAC-3′ and probe #95. Primers and probes were mixed with Brilliant probe-based QPCR Master Mix (Agilent, Santa Clara, USA) and then diluted with distilled deionized water up to 20 µl. An Mx3000P QPCR System (Stratagene, La Jolla, USA) was used for quantitative PCR. Thermal cycling conditions consisted of a 10 min template denaturation step at 95°C, followed by 50 cycles of 95°C for 30 s, 50°C for 30 s and 72°C for 30 s. Each gene expression was normalized with HGPRT mRNA content.

### Statistical analysis

Data were expressed as means ± SD. Differences between groups were evaluated for significance by one way ANOVA analysis. All tests were performed using SigmaStat v3.5 (Systat-Software, Erkrath, Germany). A p-value below 0.05 was considered statistically significant.
